# "Off/On” Fluorescent Probe based on Aggregation-Induced Quenching of ZnO-Quantum dots for Determination of Ara-C: Pharmacokinetic Applications, Adsorption Kinetics & Green Profile Assessment

**DOI:** 10.1007/s10895-023-03359-0

**Published:** 2023-08-11

**Authors:** Marwa R. El-Zahry, Rania S. Ibrahim, Hanaa M. Abd El-Wadood, Horria A. Mohamed

**Affiliations:** 1 Pharmaceutical Analytical Chemistry Department, Faculty of Pharmacy, Assiut University, Assiut, 71526 Egypt; 2 Pharmaceutical Chemistry Department, Faculty of Pharmacy, Badr University in Assiut, 2014101 Assiut, Egypt

**Keywords:** ZnO-quantum dots, Thiol-modified magnetite solid-phase micro-extraction, “Off/On” fluorescent sensor of Ara-C, Adsorption kinetics, Greenness assessment

## Abstract

Herein, a turn “Off/On” fluorescence probe based on ZnO quantum dots (ZnO-QDs) has been proposed and successfully utilized for the determination of Ara-C (cytarabine) using ceric ions (Ce^4+^) as quencher and ethylenediamine (ED) as a linker. The probe is based initially on the quenching effect of Ce^4+^ ions on the strong native fluorescence of ZnO-QDs forming the Turn Off system (Ce@ZnO-QDs) that believed to occur due to the aggregation-induced quenching (AIQ) mechanism. The second step is the addition of Ara-C in the presence of ethylenediamine (ED) that encourages the formation of Ara-C/ED/Ce^4+^ as well as the release of the free ZnO-QDs, leading to the recovery of the fluorescence intensity. The developed sensing platform shows a linear response towards Ara-C over the range of 10 to 1000 ng mL^−1^ giving a limit of detection (LOD) and limit of quantitation (LOQ) of 1.22 ng mL^−1^ and 3.70 ng mL^−1^, respectively. A dispersive magnetic solid phase micro-extraction (dMSPE) method was developed and optimized for the extraction of Ara-C in spiked human plasma using thiol-modified magnetite nanoparticles (S-MNPs). The proposed platform exhibits good sensitivity toward Ara-C in the presence of different interfering substances. Excellent recoveries are obtained after spiking different concentrations of Ara-C into rabbit plasma samples. The validated experimental parameters have been successfully applied to monitor the pharmacokinetic profile of Ara-C in rabbit plasma. A detailed adsorption kinetics study has been carried out to provide a deep insight into the adsorption behavior of Ara-C on the thiol-doped-magnetite nanoparticles. The greenness assessment of the proposed method was achieved and compared with other reported methods using two tools of greenness; the green analytical procedure index (GAPI) and the analytical greenness calculator AGREE.

**Supplementary Information:**

The online version contains supplementary material available at 10.1007/s10895-023-03359-0.

## Introduction

Ara-C (cytarabine), 4-amino-1-β-D-arabinofuranosylpyrimidin-2(1H)-one, is one of the chemotherapeutic that belong to the group of antimetabolites, pyrimidine nucleoside analogues, that used to treat acute myeloid leukemia together with anthracycline drug, acute lymphoblastic leukemia and prophylaxis of meningeal leukemia and chronic myeloid leukemia. It is also used in treatment of Hodgkin's and non-Hodgkin's lymphoma. Besides its chemotherapeutic effect, Ara-C has antiviral and immunosuppressant properties [1].

It was reported that Ara-C is not effective orally due to its rapid de-amination in the gastrointestinal tract, so it can be given only by intravenous injection (I.V.), intravenous infusion, subcutaneously and intrathecally [1]. As far as the literature survey, the reported methods for Ara-C determination include spectrophotometry [2–4], chemiluminescence [5], electrochemical [6–9], and radioimmunoassay [10, 11].

Different chromatographic techniques were also reported for Ara-C determination; including micellar electrokinetic chromatography [12, 13], gas chromatography [14] and liquid chromatography either alone or in combination with other drugs [15–24]. Among all the previous reported methods, the fluorimetric method exhibited an extensive importance owing to its simplicity, real time monitoring, and non-destructive operation. After careful survey, it can be concluded that the proposed research is the first trial discussing a fluorimetric probe for determination of Ara-C in pharmaceutical preparation and real rabbit plasma utilizing ZnO-QDs as a fluorescent platform for sensing.

Different types of quantum dots such as carbon [25], graphene [26], and metals [27] have been reported for determination of different biological compounds. Among these different types, ZnO-QDs have been widely applied in many fields including bio-imaging or bio-labeling, bio-sensing, immunosensing, drug delivery, sensors, photoluminescence sensor, and electrochemical sensors [28, 29].

**Scheme 1:**
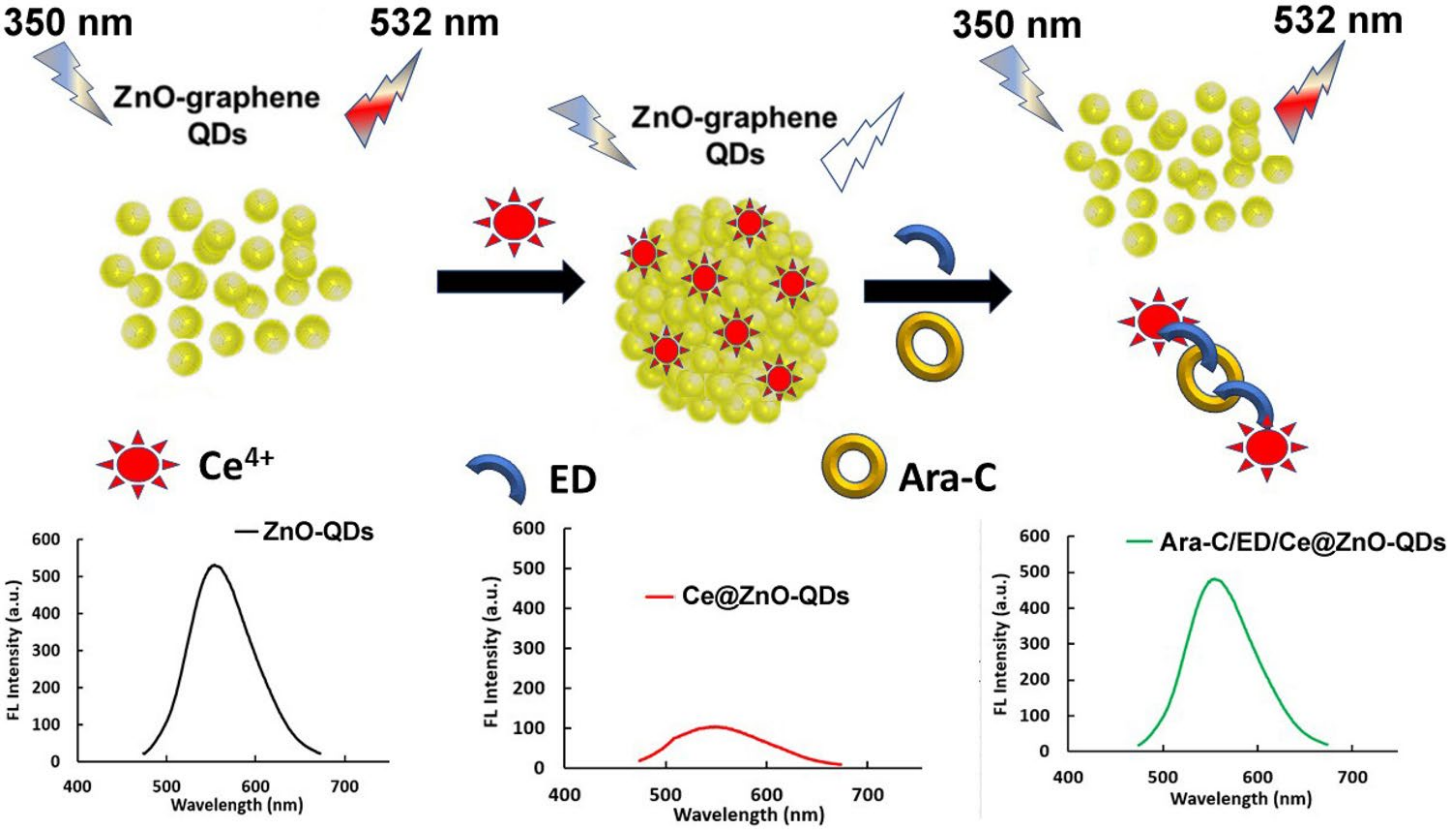
A graphical presentation of Turn Off/ON strategy of ZnO-QDs with Ce^4+^

Quantum dots have been attracted the attention of scientists owing to its high quantum yield, low toxicity, excellent photo-stability, high sensitivity and selectivity, good bio-compatibility, excellent chemical inertness, tunable luminescence, and resistance to photo bleaching. Recently, there have been studies of the aggregation-induced quenching of the fluorescence of QDs provided by divalent cations Ca^2+^; which is attributed to electrostatic screening of the CaCl_2_ electrolyte [30]. Additionally, the quenching of Mg^2+^ and Al^3+^ was reported to be attributed to the binding of the metal to the ligands [31]. Among these metal ions, cerium (Ce), that has been selected for the proposed probe as a strong quencher of ZnO-QDs through photo-induced electron transfer and aggregation induced quenching mechanism. The results show an interesting Off/On fluorimetric probe with the stepwise addition of Ce^4+^ and Ara-C (Scheme 1). While, the use of ZnO-QDs as sensing platform of Ara-C has received less attention. It’s noteworthy that Ce^4+^ ions have a great affinity for the aggregation of the prepared QDs leading to quenching. After the addition of Ara-C, ethylenediamine (ED) acts as a linker between Ce^4+^ and Ara-C forming a stable complex. This provides a great motivation to develop the fluorescence sensing probe for the analysis of Ara-C using ZnO-QDs.

In order to increase the detection power of the developed method, dispersive magnetic solid phase micro-extraction (dMSPE) technique was applied prior to the analysis in the real samples to decrease the limit of detection. Functionalized nano-magnetic particles were used owing to their surfactant features in addition to magnetic features which facilitate the separation from aqueous solution by applying an external magnetic field [32]. Magnetic separation is significantly more convenient, cost-effective, and efficient, avoiding time-consuming operation such as filtration or centrifugation. Such advantages make dMSPE as a promising alternative technique for selective and sensitive sample extraction and clean-up [33].

At the beginning of 1990s, the term of green chemistry has been emerged and became a tool of providing sustainable development in laboratories. The basic concept of green chemistry provides the need of reduction of using reagent and auxiliaries and elimination of the use of solvent and the generation of hazardous substances [34].

Additionally, lowering the energy consumption by using mild reaction conditions and avoiding derivatization. There are 12 criteria proposed by Anastas [35]. The main idea is to compare the greenness of existing reported methods with newly developed one by the development and application of measurement procedures. Now, there is a variety of greenness measurement tools, however the most common are the green analytical procedure index (GAPI) and AGREE tool. GAP index has a distinct advantage of covering the whole steps of the analytical method.

In the present study, thiol doped-magnetite nanoparticles (S-MNPs) have been utilized and dispersed into the sample to achieve separation. This disperse mode provides a high contact surface area between the modified MNPs and the analyte to remove the matrix components that interfere the detection of analyte.

A detailed study of the adsorption behavior of Ara-C on the surface of the modified magnetic NPs (S-MNPs) has been conducted to determine the kinetic model and the adsorption isotherm that best describe the adsorption of Ara-C. To achieve this, adsorption capacity was measured as a function of contact time.

Herein, a fluorescent turn “Off–On” probe based on ZnO-QDs coupled with dMSPE purification protocol has been developed for highly selective and sensitive determination of Ara-C. The probe strategy is based on the strong native fluorescence of ZnO-QDs which shows fluorescence quenching towards Ce^+4^ (Turn Off) forming Ce@ZnO-QDs. Upon addition of ED as a linker followed by Ara-C, the fluorescence was recovered (Turn On) and utilized for the sensitive determination of Ara-C in pharmaceutical dosage forms and spiked human plasma. However, the use of ZnO-QDs as the fluorescent probe for the turn Off/On strategy of Ara-C has not been reported yet.

Based on the magnetic purification and the fluorescence probe, the proposed platform was employed for the first time to study the pharmacokinetic of Ara-C in rabbits plasma samples showing wide detection range and good selectivity.

## Experimental

### Chemicals & Reagents

Ara-C (cytarabine, 98% purity) was purchased from AK scientific (California, USA). Tabine ampoules (100 mg/5 ml) is obtained from the local Egyptian market (Hikma pharmaceuticals, Cairo, Egypt). Ammonium ceric sulphate (LYCHE company, Mumbai- India), Citric acid anhydrous, used for preparation of graphene quantum dots, (EL- Nasr Pharmaceutical chemicals CO., Cairo, Egypt), Sodium hydroxide (Alpha Chemicals, Cairo, Egypt), Acetonitrile 99.5% extra pure, Alpha chemicals) were obtained.

Human plasma sample was obtained from Assiut University Hospitals (Assiut, Egypt).

### Instruments

Shimadzu RF-5301 PC spectroflourimeter (Tokyo-Japan) with 1-cm quartz cells used for spectroflourimetric measurements, Sartorious handy balance-H51 (Hannover-Germany), FALC vortex -MIX (FALC instruments-Italy), Laboratory centrifuge (Bremsen ECCO- Germany), hot plate with stirrer (stuart CB 302, bibby scientific, UK), Transmission electron microscope (JEOL JEM-100 CXII, Japan) instrument, Fourier Transform infrared spectrometry (Nicolet IS 10, USA), UV–visible spectrophotometer (Thermo evolution 300 UV–VIS, England), X-ray diffraction (XRD), PW 2103 Philips diffractometer (λ = 1.5418 Å) operated at 35 kV and 20 mA stimulated by Cu Kα radiation (Ni‐filtered) over the range 10–80° (2*θ*).

### Synthesis of ZnO-QDs

Briefly, 0.46 g of Zn(CH_3_COO)_2_ and 0.02 g of NaOH were weighed and dissolved in 30 mL and 10 mL of ethanol, respectively. Then, the alkaline solution was added to the Zn^2+^ solution with continuous stirring in an ice bath. After 20 min, the ZnO-QDs were obtained. The obtained QDs were washed several times with ethanol and freeze dried to get the QDs powder that stored for subsequent applications [36].

### Synthesis of S-MNPs

#### Synthesis of Magnetite Nanoparticles (MNPs)

In three necked flask, 3.0 g of FeCl_3_·6H_2_O and 1.0 g of FeSO_4_·7H_2_O were weighed and dissolved in 80 mL water. The mixture was kept at 80 ℃ with continuous stirring. After 20 min., 25 mL of ammonia was added to the mixture dropwise. A black precipitate of MNPs was obtained and washed with water and ethanol for three times, followed by drying at ambient temperature [37].

#### Synthesis of Thiourea-formaldehyde Compound

Typically, 1.5 g thiourea was dissolved in 10 mL methanol, followed by addition of 1.5 mL formaldehyde, the pH value was adjusted to 3.0. Then, the mixture was refluxed at 90°C, until a white viscous product was obtained. The obtained product was washed three times with methanol.

#### Synthesis of Thiol-modified Magnetic Nanoparticles (S-MNPs)

In order to prepare the S-MNPs, one gram of the magnetite and 0.1 g of thiourea-formaldehyde were dissolved in 50 mL of distilled water, followed by 24 h stirring and then rinsing with distilled water.

#### Magnetic Solid-phase Extraction Procedure

Magnetic-solid phase extraction procedure (MSPE) was processed through thiol-modified magnetic nanoparticles (S-MNPs) as sorbent to extract Ara-C from spiked human plasma. The supernatant obtained after deproteinization were completed to 20 ml with Britton-Robinson buffer (pH 4.0). An amount of 15 mg of S-MNPs was added and vortexed for 15 min. to ensure full adsorption of Ara-C on the external surface of S-MNPs. After that, an external magnet was employed to separate S-MNPs from the solution mixture followed by decantation of the supernatant and elution of the analyte via 0.5 ml methanol by sonication for 5 min. The solution was then separated by placing an external magnet, followed by collecting the supernatant for further analysis.

#### Fluorescence Sensing Procedure

Into a 10 ml volumetric flask, 1 mL of ZnO-QDs and 2 mL of 10^–3^ mol L^−1^ ceric ammonium sulphate were added. One milliliter of ethylenediamine followed by 1.0 mL of standard or sample solution of Ara-C were added to this mixture and completed to the mark with distilled water. All the fluorescence emission spectra were recorded at room temperature with λ_exc._ at 532 nm and λ_em._ at 350 nm against reagent blank.

## Results & Discussion

### Characterization of ZnO-Quantum Dots (ZnO-QDs)

**Fig. 1:**
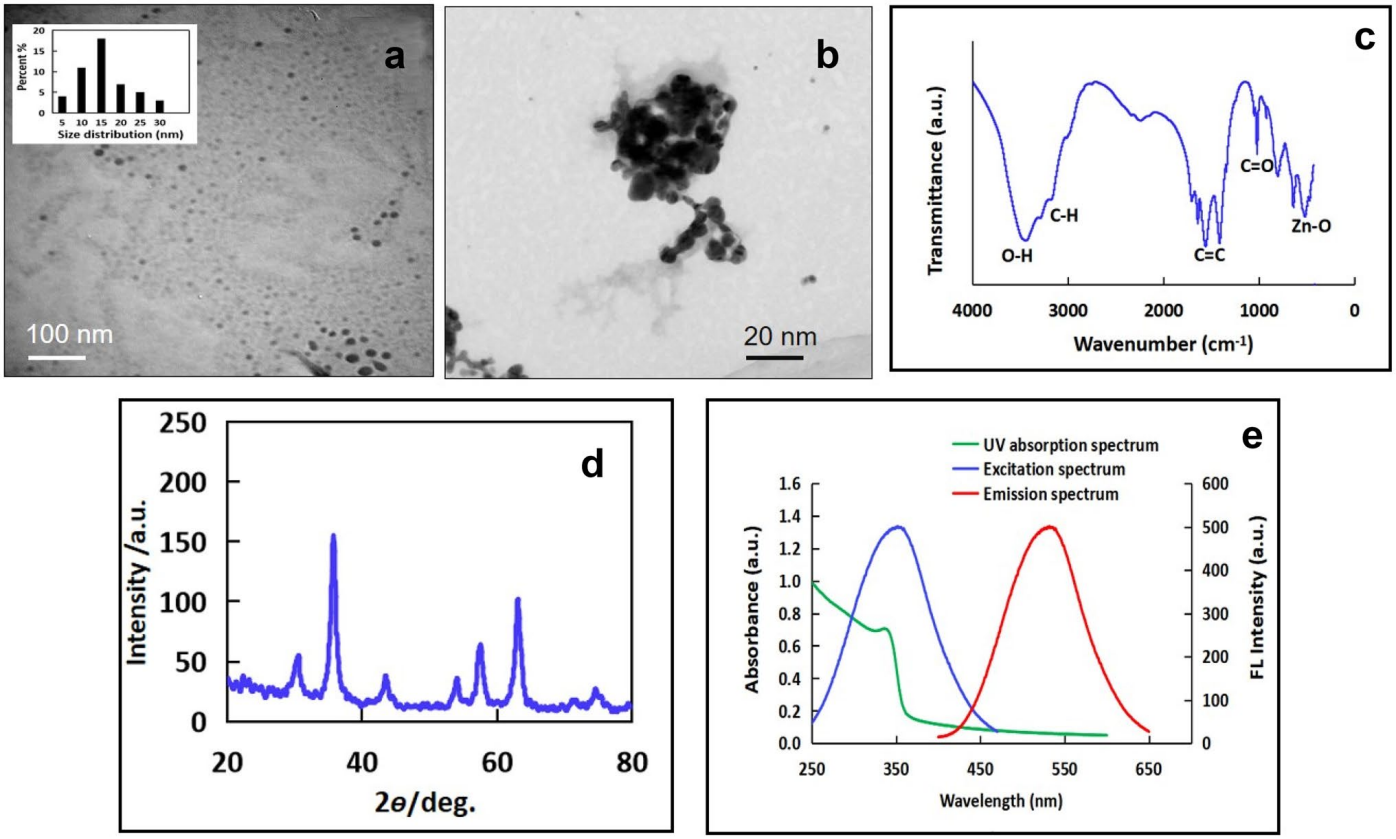
**a** TEM image of ZnO-QDs with particle size distribution, **b** TEM image of the aggregated ZnO-QDs on addition of Ce^4+^, **c** FT-IR spectrum, **d** p-XRD spectrum, and **e** UV–VIS and fluorescence spectra of the as-prepared ZnO-QDs

Different methods were employed for characterization of the prepared ZnO-QDs. Figures 1a, b represent TEM images ZnO-QDs and their aggregates, respectively. It revealed that the prepared ZnO-QDs have a fairly uniform size with good dispersion as well as no large agglomerates. The sample shows Gaussian distribution with an average size of 15.0 nm (Inset of Fig. 1a).

Figure 1b shows the formation of large disordered ZnO-QDs aggregates without morphological change when Ce^4+^ ions were added which may be attributed to the ground-state electronic coupling or energy transfer through aggregates [38].

It is worthy to mention that the change is only in the aggregation state, but the single unit of quantum dots had not changed, indicating that the Ce^4+^ induced FL quenching may be attributed to the aggregation induced quenching (AIQ).

In order to identify the characteristic peaks of the functional groups on the prepared QDs, FT-IR spectrum was recorded. As shown in Fig. 1c, the vibrations of OH, CH, and C = C are located around 3500, 3000, and 1600 cm^−1^, respectively. The vibration band assigned for ZnO is located at 533 cm^−1^. These FT-IR observations confirmed the successful synthesis of ZnO-QDs and well-matched with the previous reported studies [39].

The crystallinity of the as prepared ZnO-QDs, another important factor, was investigated using powder X-ray diffraction (pXRD). As shown in Fig. 1d, the pXRD pattern indicates high crystallinity, which can be identified as (100), (002), (101), and (102) plans. The obtained results were well matched with previous reported study [40].

In order to study the optical properties of ZnO-QDs, the UV–VIS absorption and the fluorescence emission spectra are shown in Fig. 1e. The absorption peak at 350 nm can be appointed to the n-π^*^ transitions of C = O functional group. Additionally, the ZnO-QDs exhibit strong FL emission at 532 nm when irradiated at 350 nm. This emission peak is mainly assigned to the oxygen vacancies [41].

The effect of excitation wavelength on the emission fluorescence wavelength was investigated from 290 to 400 nm (Fig. S1). It can be depicted that the emission fluorescence intensity reached the maximum with excitation wavelength at 350 nm.

### Quenching Mechanism of Ce^4+^ Ions on the FL Intensity of ZnO-QDs

The effect of Ce^**4+**^ concentration on the native FL intensity of the prepared ZnO-QDs was investigated. As shown in Fig. S2, the addition of various concentrations of Ce^**4+**^ ions ranging from 2.5 to 30 μmol L^−1^ clearly decreased the native FL intensity of ZnO-QDs. To better understand the quenching behavior, FL spectra were monitored along with a binding constant and different Ce^4+^ concentrations. The binding constant was calculated using Stern–Volmer equation.

$${F}_{0}/F=1+{K}_{sv}\left[\mathrm{Q}\right]$$

where, F_0_ and F are the fluorescence intensities of ZnO-QDs at 532 nm in the absence and presence of Ce^4+^ free solution, respectively. **Q** is the Ce^4+^ concentration, while *K*_*sv*_ is the Stern–Volmer quenching constant. If a linear plot between FL ratio (F_0_/F) and **Q** is obtained, the Stern–Volmer statement of binding of Ce^4+^and ZnO-QDs is valid. Stern–Volmer quenching constant (*K*_*sν*_), which is a measurement tool of the quenching efficiency of the quencher, generally shows more sensitive system with a steeper slope and consequently a higher K_Sν_ value that was calculated to be 0.09 × 10^4^ mol^−1^ with a high correlation coefficient (R = 0.9984). To investigate the type of quenching mechanism, the relationship of FL ratio and **Q** concentration of Ce^4+^ at different temperatures was monitored and recorded in Fig. S3. The Stern–Volmer plots show good linearity between F_0_/F and **Q** concentration of Ce^4+^ over the studied range providing slopes to be **3.751** at 298 K, **2.902** at 308 K and **2.366** at 318 K, respectively with a correlation coefficient (R^2^) higher than 0.990. It can be inferred that the obtained *K*_*sv*_ values decreased with increasing the temperature from 298 to 318 K suggesting that the quenching mechanism is static quenching [42].

### Detection of Ara-C by ZnO-QDs Based Sensing Probe

**Fig. 2:**
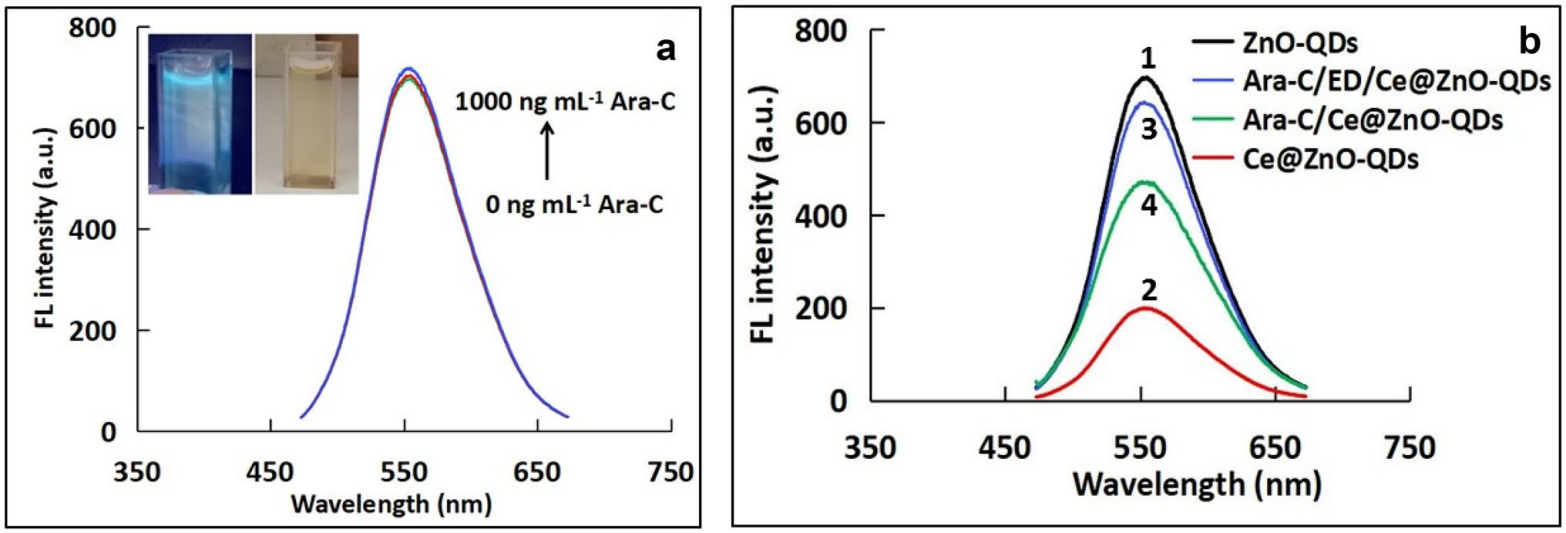
**a** FL spectra of ZnO-QDs in the presence of different concentrations of Ara-C. Inset: photographs of ZnO-QDs taken under visible light (right) and UV light (left), **b** The FL spectra of (1) ZnO-QDs, (2) Ce^4+^@ZnO-QDs, (3) Ara-C/ ED/ Ce^4+^@ZnO-QDs, (4) Ara-C/Ce^4+^@ZnO-QDs (Ara-C concentration 100 ng mL^−1^)

To investigate the ability of ZnO-QDs as an optical sensor, different concentrations of Ara-C have been added to ZnO-QDs system, and the change in the FL intensity was monitored. As shown in Fig. 2a, the FL intensity remains unchanged upon addition of 0 -1000 ng mL^−1^ Ara-C, confirming that Ara-C has no interaction with the bare ZnO-QDs. As shown in Fig. 2b, the FL intensity of ZnO-QDs has been greatly quenched after the addition of Ce^4+^ ions (Ce@ZnO-QDs). After the addition of Ara-C to Ce@ZnO-QDs system, the FL was restored but incompletely due to the formation of Ara-C/Ce complex. The results shown clearly indicate that the FL intensity cannot be completely recovered without the addition of ED. This may be assigned to the low complexation affinity of Ara-C toward Ce^4+^ions.

Compared with the Ara-C/Ce@ZnO-QDs; the addition of ED as a linker forming Ara-C/ED/Ce@ZnO-QDs system greatly recovered the Fl intensity. In the proposed system (Fig. 2b), more than 90% of the FL intensity of ZnO-QDs has been recovered upon addition of 100 ng ml^−1^ Ara-C to ED/Ce@ZnO-QDs probe.

Figure 2b shows the change of the FL intensity before and after addition of Ara-C, which clearly demonstrate the turn Off/On strategy for determination of Ara-C using ED/Ce@ZnO-QDs probe as label-free tool. It’s worth to mention that the addition of ED doesn’t affect the fluorescence properties of Ce@ZnO-QDs, but shows its senior complexation ability as a ligand to bridge Ce^4+^ and Ara-C, leading to formation of stable complex Ce/ED/Ara-C followed by the release of ZnO-QDs to restore the native fluorescence. These results can be explained via the high affinity of the primary amine group in Ara-C toward ED/Ce more readily than ZnO-QDs, and eventually leads to the release of ZnO-QDs and restoring the fluorescence. These results confirm the exceptional properties of the proposed sensing probe of Ara-C using Ce^4+^ as a fluorescence quencher and ED as a linker between Ce^4+^ and Ara-C molecules [43, 44].

### Optimization of Fluorimetric Probe Variables

The influence of different parameters on the FL intensity of QDs; such as type of quencher, Ce^4+^ ions concentration, ZnO-QDs concentration, reaction and stability time, and diluting solvent were examined.

### Effect of Type of Quencher

**Fig. 3:**
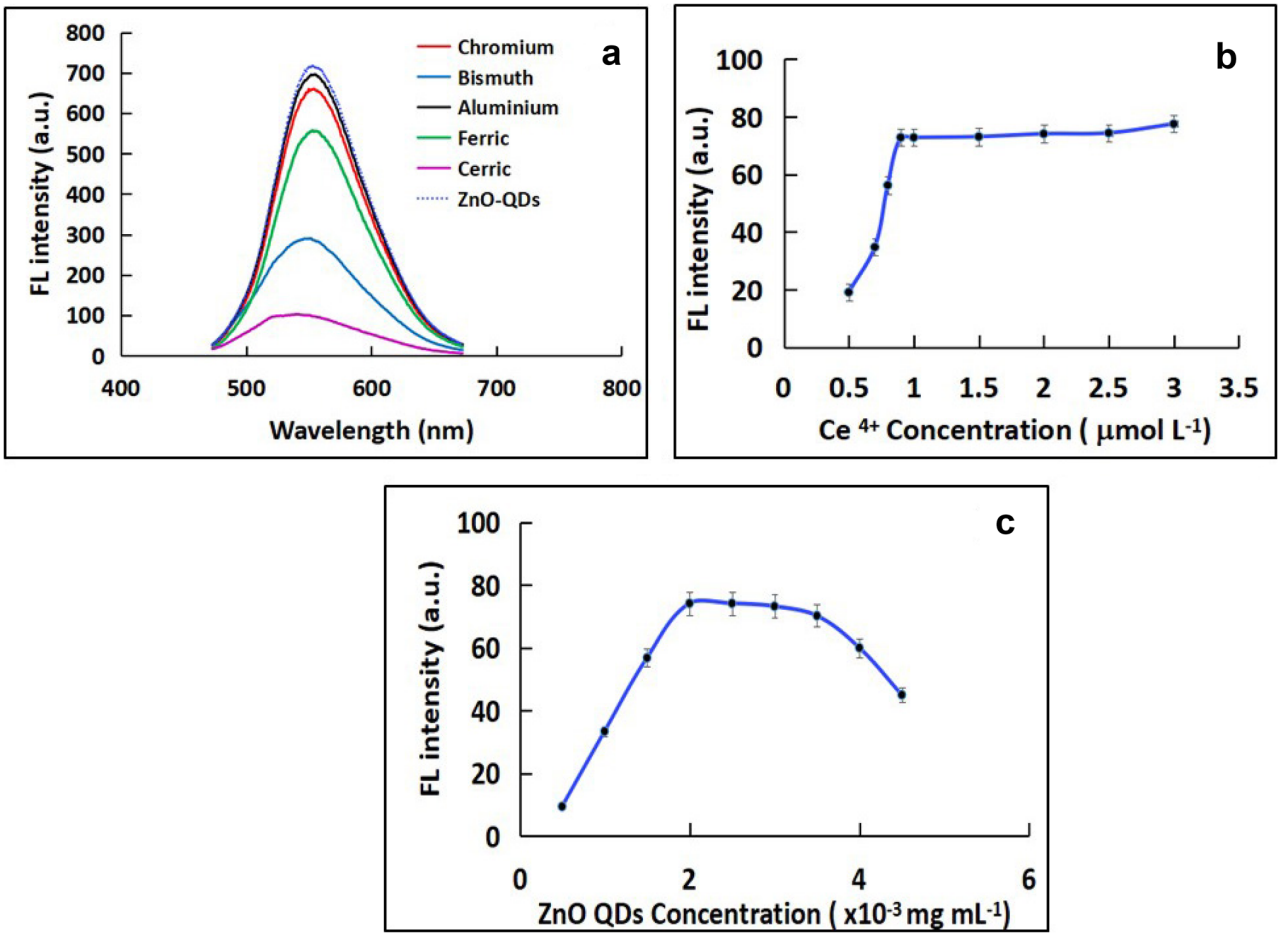
Effect of **a** Type of quencher, **b** concentration of Ce^4+^, and **c** concentration of ZnO-QDs on FL intensity of 100 ng mL^−1^ Ara-C at ʎ_exc_= 350 nm and ʎ_em_= 532 nm

Different cations were investigated as a quencher for the native fluorescence of ZnO-QDs. Figure 3a shows the change in the FL intensity with the change of the type of quencher. As depicted, ceric ions exhibit the strongest quenching response of the FL intensity of the as-prepared ZnO-QDs. So, it is selected to be used as a quencher in the developed system.

### Effect of Ce^4+^Concentration

In order to study the impact of Ce^4+^ ions on the FL intensity of sensing system, different concentrations of Ce^4+^ ions were investigated over the range of 0.5 to 3 μmol L^−1^. As shown in Fig. 3b, the FL intensity increased by increasing the concentration of Ce^4+^ until it reaches 1.0 μmol L^−1^ after that it remains stable with increasing concentrations of Ce^4+^. So, 1.5 μmol L^−1^ is selected to be the optimum concentration of Ce^4+^ used in the developed probe.

### Effect of ZnO-QDs Concentration

The effect of the as-prepared ZnO-QDs concentration on FL intensity was investigated. As it shown in Fig. 3c, the FL intensity was increased with increasing ZnO-QDs concentration. On the other hand, the continues increase of the studied QDs concentration may result in self-quenching of the QDs fluorescence [41] leading to a decrease in the FL intensity. Based on these factors, a concentration of 2.5 × 10^–3^ mg ml^−1^ ZnO-QDs was selected as an optimum concentration for the subsequent work.

### Effect of Reaction and Stability Time

The influence of reaction time on the FL intensity was investigated. The reaction time between Ara-C and Ce@ZnO-QDs system was studied over the time ranged from 0 to 30 min. It was observed that the FL intensity did not change significantly upon increasing the reaction time.

The stability of the restored FL of the studied QDs followed the addition of Ara-C to the developed system ED/Ce@ZnO-QDs was monitored over the time range 0 to 25 min. It was noticed that the FL intensity of the released QDs remained stable with time.

### Effect of Diluting Solvent

Different polar and non-polar solvents such as double distilled water, methanol, ethanol, acetonitrile and dimethylformamide (DMF) were applied as diluting solvents. It was observed that the highest FL intensity was recorded upon using double distilled water, while methanol, ethanol and acetonitrile gave a turbidity after their addition to the reaction solutions. Additionally, it was observed that severe turbidity was seen upon addition of DMF as diluting solvent. Hence, double distilled water was selected as diluting solvent for subsequent experiments, confirming the greenness of the proposed method.

### Optimization of Dispersive Magnetic Solid Phase Micro-extraction Procedure

In order to remove the interference of the complicated biological matrices, such as plasma and urine samples, a purification method based on the dMSPE is optimized. The proposed method is based on thiol-functionalized magnetic NPs (S-MNPs) as sorbent. The adsorption process of Ara-C molecules on the S-MNPs surface may occur via the formation of hydrogen bonds between the hydroxyl groups of ribose ring and the amine groups of Ara-C molecule and the SH- group doped on the S-MNPs surface. The investigation and optimization of the experimental variables using one variable at a time method were conducted in triplicate and the average was calculated.

### Type of Magnetic Surface Modifiers

**Fig. 4:**
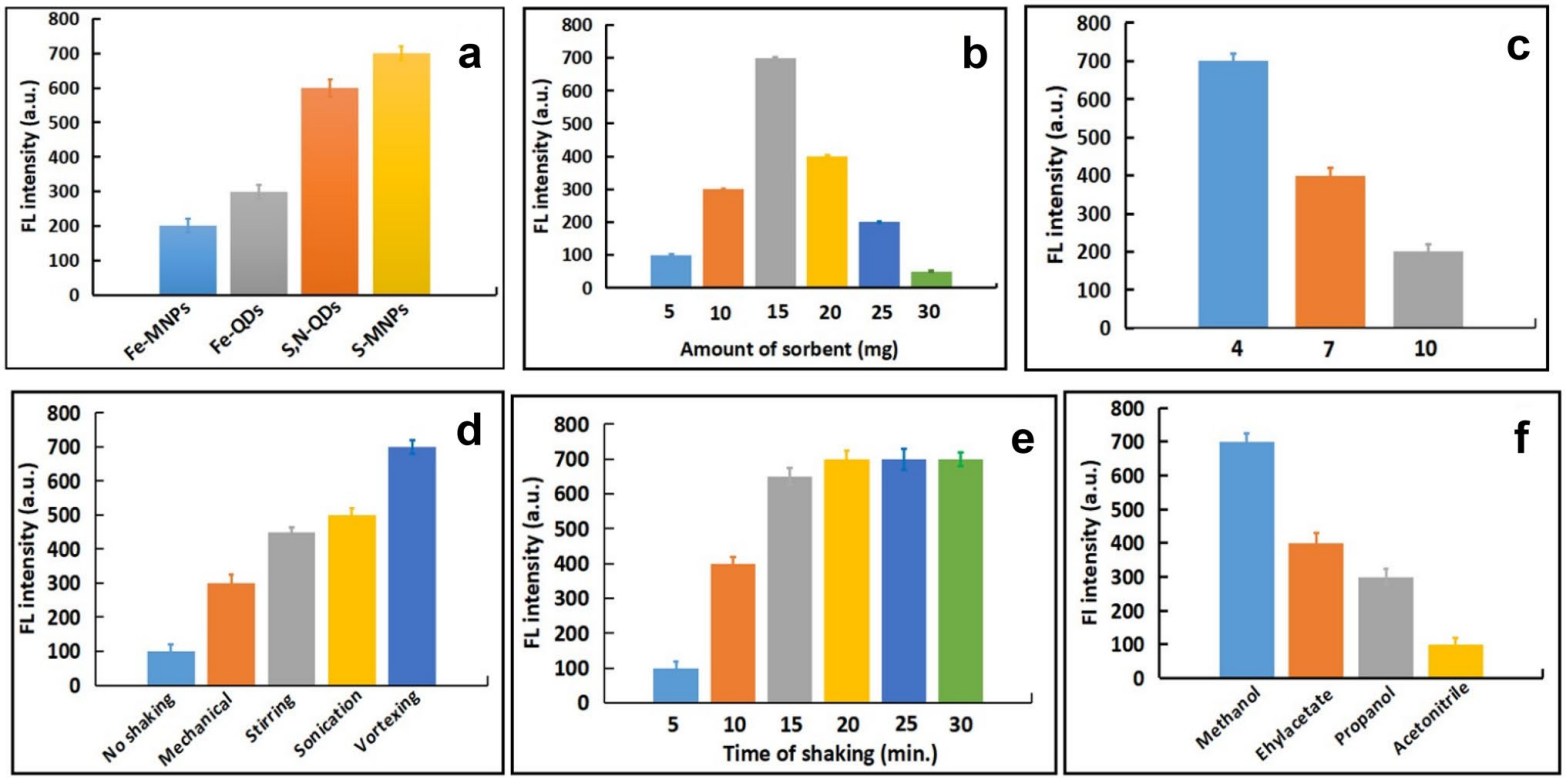
Optimization of the dMSPE **a** types of MNPs, **b** amount of S-MNPs, **c** pH, **d** type of shaking, **e** time of shaking, and **f** type of eluent solvent. The optimization experiments were carried out using 100 ng mL^−1^ Ara-C

Four different surface modifications were investigated for their extraction of Ara-C; magnetic nanoparticles (Fe-MNPs), S-MNPs, iron-quantum dots (Fe-QDs), and sulfur, nitrogen-codoped quantum dots (S, N-QDs). As shown in Fig. 4a, S-MNPs gave the highest value of the restored FL. These results may indicate the strong interaction between the modifier coating of magnetic nanoparticles and Ara-C molecules. As a result of these findings, S-MNPs were selected to be the surface modifier used for the efficient extraction of the interested analyte from the human plasma samples.

### Amount of Sorbent

While the other experimental parameters remained constant, different amounts ranged from 5 to 30 mg of the selected sorbent (S-MNPs) were applied. As described in Fig. 4b, the highest FL intensity was obtained using 15 mg sorbent. When the amount of sorbent increased till 25 and 30 mg, the FL intensity was markedly decreased which may be assigned to the agglomeration of the MNPs, leading to loss the active adsorption sites [42]. So, the optimum amount of sorbent used in subsequent extraction was selected to be 15 mg.

### Effect of pH

As Ara-C is a polar drug, its extraction procedure would be pH-depended. In order to ensure efficient extraction, pH value of the sample solution medium should be adjusted. Different pH values of Ara-C sample extraction solution were adjusted at 4.0, 7.0, and 10.0. As depicted in Fig. 4c, the highest FL intensity was observed at pH = 4.0, so it was selected as an optimum pH value. This may be due to protonation of the polar function groups (-OH of ribose ring and -NH_2_) at low pH values leading to electrostatic attraction with the modified sorbent, hence an efficient extraction of Ara-C molecules is provided at low pH.

### Type and Duration of Shaking

Different procedures of shaking; such as mechanical, sonication, stirring, vortex, and no shaking, were carried out to study its impact on the extraction efficiency of the studied analyte. As shown in Fig. 4d, the highest FL intensity was recorded upon using vortex-assisted extraction. Additionally, vortex-based method was applied at different times at 5, 10, 15, 20, 25, and 30 min. Figure 4e depicted that the highest FL was observed at time higher than 15 min. So, the optimum mixing time was selected to be 20 min.

### Type of Eluent

Different eluents were investigated to achieve an efficient elution of the adsorbed analyte from the applied sorbent. As can be depicted from Fig. 4f, the best result was provided upon applying methanol as an elution solvent. So, it was selected as the optimum elution solvent.

### Method Validation and Data Analysis

In order to validate the developed method for sensing Ara-C; linearity, precision, accuracy, and robustness were examined following the ICH (International Council for Harmonization) guidelines [45].

**Fig. 5:**
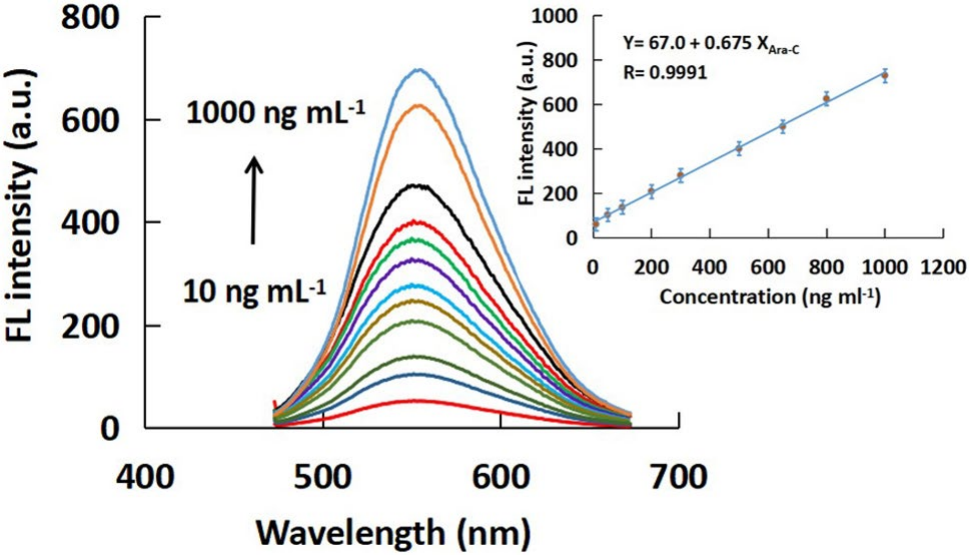
FL spectra of different concentrations of Ara-C ranged from 10 to 1000 ng ml^−1^ using the proposed system. Inset: Calibration plot of different Ara-C concentrations on ED/ Ce^+4^@ZnO-QDs

In order to manage the sensitive detection of Ara-C, different concentrations of Ara-C were added to the developed system probe. As shown in Fig. 5, under the optimum conditions, the response of FI values is concentration depended exhibiting a linear relationship between the restored FL intensity and Ara-C concentrations over the range of 10 to 1000 ng mL^−1^. Different statistical parameters, such as correlation coefficient (R), intercept, slope, limit of determination and quantitation were calculated and listed in Table 1.

**Table 1:** Statistical parameters and precision values of the determination of Ara-C using the proposed probe

**Parameters**	**The calculated value**
Linearity range (ng mL^−1^)	10—1000
Intercept ± SD^a^	67.0 ± 0.176
Slope ± SD^b^	0.675 ± 6.35 × 10^–4^
Correlation coefficient (R)	0.9991
LOD (ng mL^−1^) ^c^	2.87
LOQ (ng mL^−1^) ^d^	8.69
Intra-day precision^e^ (% RSD, n = 6^f^)	≤ 1.560
Inter-day precision^e^ (% RSD, n = 6^f^)	≤ 1.739

^a^Standard deviation of the intercept

^b^Standard deviation of the slope

^c^Limit of detection (ng ml^−1^)

^d^Limit of quantitation (ng ml^−1^)

^e^Relative standard deviation

^f^Mean values of six measurements at three different concentration levels

Repeatability of the investigated method was studied by performing intra-day and inter-day precision. It was found that RSD% values are ranged from 0.597 to 1.739. Recovery % was calculated for the measured concentrations of Ara-C and compared with the true values (Table 1). Recovery percentages were ranged from 98.82 to 101.11% indicating good accuracy of the developed method.

According to ICH guidelines, robustness of the proposed probe was investigated. Various parameters have been investigated for the robustness; such as concentration of ZnO-QDs, concentration of Ce^4+^ solution, and the excitation wavelength. The results listed in Table S1 indicate that the small variations of these parameters have no significant effect on the quantification of Ara-C. This result confirms the good reliability and robustness of the developed probe.

### Selectivity and Interference Studies

In order to evaluate the selectivity of the fluorimetric probe, the change in FL intensity of Ara-C/ED/Ce@ZnO-QDs was recorded in the presence of different metals and some interfering biological substances. The interference was checked by the addition of 100-fold excess concentration of the interfering substances and the obtained results are represented as pie chart in Fig. S4. The results indicate that the proposed platform is competent to efficiently and selectively detect Ara-C among commonly found biological and chemical species.

### Real Time Monitoring of Ara-C

To study the applicability of the fluorimetric probe as a sensing tool of Ara-C, the proposed method has been applied for sensing Ara-C in some complicated matrices, such as pharmaceutical dosage form and human plasma samples [46].

Determination of Ara-C drug in Tabine ampoules was applied and the calculated recoveries were compared with the reported method [2]. Excellent recoveries were obtained with good agreement with the claimed amount as shown in Table 2.

**Table 2:** Determination of Ara-C in pharmaceutical dosage form and spiked human plasma using the developed fluorimetric probe

Dosage form	% Recovery ± SD^a^			
	Proposed method	Reported method^c^	*t-*test value^b^	*F-*test value^b^
Tabine ampoules (100 mg 5 mL^−1^)	99.47 ± 0.88	99.08 ± 0.341	0.613	2.35
Human plasma	Added amount (ng mL^−1^)	% Recovery ± SD*		
	10 200 1000	101.71 ± 2.02 99.02 ± 0.479 99.88 ± 0.986		

^a^Average of six replicates

^b^Theoretical values at 95% confidence limit; t = 2.228, F = 5.053

^c^Ref. 2

Three concentrations of Ara-C were spiked in human plasma and the recovery percentages were calculated as shown in Table 2. The recoveries ranged from 99.02 to 101.7%, inferring that the developed probe has a wide applicability for sensing Ara-C in different complicated matrices.

An optimization procedure of different parameters that may affect the real-time applicability of the proposed probe for determination of Ara-C in human plasma; such as the type and volume of protein precipitating reagent, the speed and duration of centrifugation have been investigated and depicted in details as shown in Fig. S5.

### Pharmacokinetic Study

**Fig. 6:**
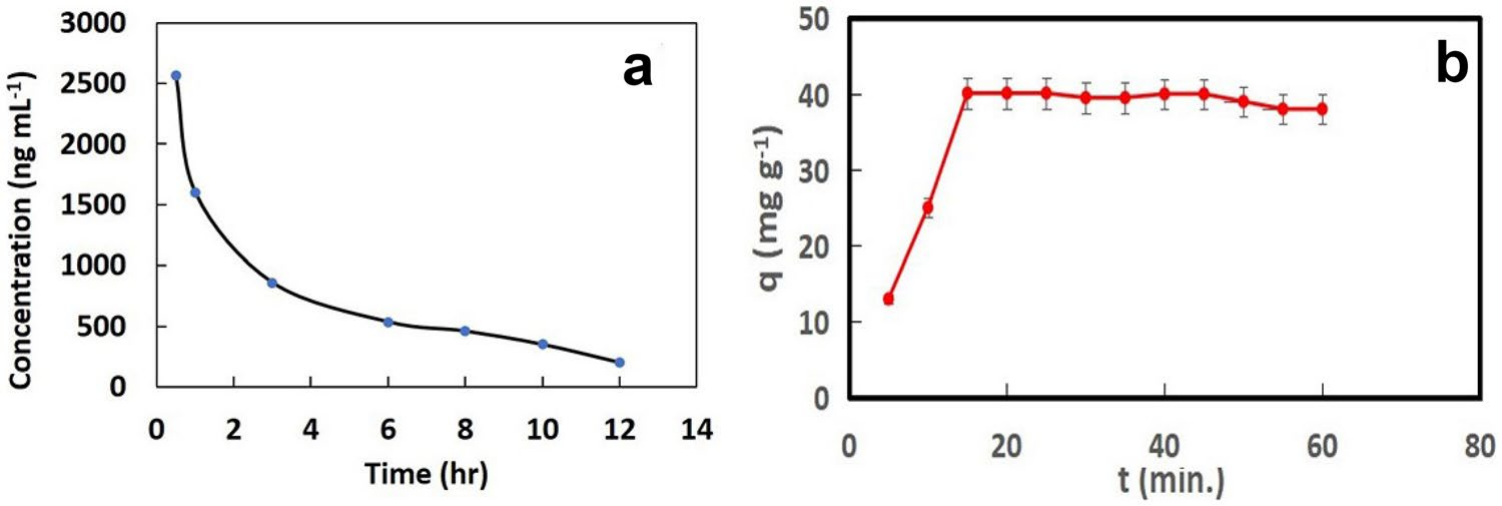
**a** The plasma concentration–time profile of Ara-C after administration of single I.V. single dose equivalent to 17 mg kg^−1^ from its pharmaceutical dosage form, **b** Adsorption capacity curve of S-MNPs towards Ara-C

The validated fluorimetric method was utilized to study the pharmacokinetics of Ara-C in rabbit plasma after I.V. injection of single dose containing 17 mg kg^−1^. The mean plasma concentration of single I.V. dose of Ara-C in rabbit plasma sample monitored by the developed sensor is shown as time profile in Fig. 6a. It was observed that the concentration of Ara-C was initially high, followed by a decrease with increasing time until it becomes constant after 12 hr of injection.

The mean pharmacokinetic parameters were calculated using Kinetica 5.0 software, listed and compared with previously reported work as shown in Table 3 [47].

**Table 3:** Mean pharmacokinetic parameters of Ara-C after I.V. injection of Ara-C in rabbit plasma

Pharmacokinetic parameter	The proposed method	The reported method^a^
t_½_ (hr)	3.47 ± 0.425	1.86 ± 0.719
Distribution volume V_d_ (L)	12.69 ± 0.336	–––
Clearance Cl (L hr^−1^)	2.73 ± 0.214	–––
AUC (µg. hr mL^−1^)	131.2 ± 15.4	26.29 ± 4.11
C_max_ (µg mL^−1^)	2.515 ± 2.55	16.85 ± 3.50

^a^Ref. [47]

### Comparison the Proposed Method With other Reported Methods

As shown in Table 4, the analytical performance of the proposed method compared with different chromatographic, spectrophotometric, and chemiluminescence probes toward Ara-C is presented. The table shows that the proposed probe exhibited a wide linear range at lower LOD value, indicating higher sensitivity and superior ability toward Ara-C analysis.

**Table 4:** Comparison of the proposed system with some previously reported methods for determination of Ara-C

Technique	Linear range	LOD	Ref
UV Spectro-photometry	10–50 μg mL^−1^	0.71 μg mL^−1^	[3]
chemiluminescence	6.0 × 10^–9^ –1.0 × 10^–7^ mol L^−1^	7.6 × 10^–10^ mol L^−1^	[5]
Electrochemical biosensor	0.01–90 μmol L^−1^	2.8 nmol L^−1^	[8]
Potentiometric	1.0 × 10^−6^–1.0 × 10^−3^ mol L^−1^	5.5 × 10^−7^ mol L^−1^	[9]
UPLC	25–150 μg mL^−1^	0.64 μg mL^−1^	[17]
LC–MS/MS	20–300 ng mL^−1^	–––	[22]
RP-HPLC	2–10 μg mL^−1^	0.36 μg mL^−1^	[23]
The proposed probe	10–1000 ng mL^−1^	2.87 ng mL^−1^	This work

### Adsorption Studies

The adsorption behavior of Ara-C onto the modified S-MNPs has been investigated as a function of time by monitoring the change in Ara-C concentrations at specific intervals from 0 to 60 min. using a fixed amount of S-MNPs (15 mg) at pH 4.0.

Adsorption capacity (*q*_*t*_) of the modified S-MNPs was determined using the following mathematical equation:

$${q}_{t}=\frac{\left(Ci-Cf\right) V}{m}$$

where C_i_ and C_f_ are the initial and final drug concentration, respectively. V is the volume of drug solution; m is the mass of surface modified MNPs (g) and *q*_*t*_ is the adsorption capacity (mg g^−1^) of drug per unit adsorbent at time t (min).

Figure 6b shows the relation-ship between the adsorption capacity of the cited drug in the equilibrium solution at constant temperature and the contact time using Ara-C initial concentration of 500 ng ml^−1^. As a general view, the adsorption capacity increases with increasing the contact time until no significant change of the capacity is observed. It can also be noticed that a high initial removal was observed from the initial time up to 15 min., followed by a slower removal until a plateau is reached. This can be explained as the high availability of the adsorption active sites at the beginning  of the adsorption process. However, as more adsorption is carried out, the sites become gradually filled [48].

The collected data from the adsorption capacity was utilized to quantitatively describe the adsorption kinetics and the rate limiting step of Ara-C adsorption. To provide an individual assumption about the nature of the adsorption process of Ara-C towards the S-MNPs, pseudo first-order (PFO) and pseudo second-order (PSO) kinetic models were investigated. Pseudo first-order kinetic model is expressed by the following equation:

$$Ln\left({q}_{e}-{q}_{t}\right)=Ln {q}_{e}-{k}_{1}t$$

where q_e_ (mg g^−1^) and q_t_ (mg g^−1^) are the adsorption capacity at equilibrium state and at t time respectively, while K_1_ is the first-order rate constant, while PSO kinetic model can be expressed by the following equation:

$$\frac{t}{qt}=\frac{1}{k2 q2e}+\frac{t}{qe}$$

where K_2_ is the second-order rate constant. The linearized kinetics model plots for the adsorption of Ara-C are presented in Fig. S6. As shown in Fig. S6a, the adsorption kinetics PFO model is irrelevant to the adsorption of Ara-C (R^2^ < 0.9), while adsorption of Ara-C is better fit to PSO model providing a higher value of R^2^ (= 0.991) Fig. S6b. It can be inferred that the overall adsorption rate is controlled by the surface-reaction kinetic step and reaction order is two with respect to the adsorption active sites, meaning that each adsorbed molecule attached with two active sites of S-MNPs [49].

To provide more insightful view about the adsorption mechanism and to understand the equilibrium relation-ship between Ara-C and S-MNPs, we studied the adsorption isotherms. Adsorption isotherm can be classified according to the number of factors that affect the adsorption system into one-parameter, two-, three-, four- and five-parameter isotherm models. In this method, two-parameter adsorption isotherm models namely Langmuir (LM) and Freundlich models (FM) are studied and depicted in Fig. S7.

Langmuir isotherm assumes that the adsorption of analyte onto adsorbents carries out via the formation of monolayer of the adsorbent molecules [50]. The Langmuir isotherm can be expressed by the following equation:

$$\frac{Ce}{qe}=\frac{1}{qmax\; kL}+\frac{ce}{qmax}$$

where C_e_ is concentration of adsorbate at equilibrium (mg g^−1^). K_L_ is Langmuir adsorption constant (l. mg^−1^), which can be correlated with the change of the available surface area and porosity of the adsorbent. q_max_ (mg g^−1^) and q_e_ (mg g^−1^) is maximum adsorption capacity, and the amount of adsorbed drug per unit mass of adsorbent at equilibrium, respectively. Considering this equation, the linear plot between Ce/qe and Ce is monitored and analyzed (Fig. S7a), and R^2^ value and 1/q_max_ were estimated. The value of correlation coefficient (R^2^) was 0.9833, indicating that this model can be used to precisely describe the adsorption behavior of Ara-C onto the S-MNPs surface. The monolayer adsorption (q_max_) was estimated as 40.1 mg g^−1^ which depends on the pore size of the adsorbents. As small pore size of the adsorbent does not permit the penetration of the drug molecule into the internal pores, so the adsorption is carried out outside the pores [50].

In comparison to Langmuir, the Freundlich adsorption model assumes the repulsion between the adsorbed drug molecules. Freundlich isotherm can be expressed by the following equation:

$$\mathrm{log}\;{q}_{e}=\mathrm{log}\;{K}_{F}+\frac{1}{n}\mathrm{log}\;{C}_{e}$$

where K_F_ and n are the Freundlich adsorption isotherm constants.

Considering this equation, a linear relationship of *log q*_*e*_ and *log C*_*e*_ is the provided. The R^2^ and 1/n values were calculated as 0.8439 and 0.34, respectively, which indicate the presence of heterogeneous adsorption (Fig. S7b).

### Assessment of Greenness of the Analytical Method

In order to assess the greenness of the analytical method, two approaches have been introduced; the green analytical procedure index (GAPI) and the analytical greenness calculator (AGREE).

**Fig. 7:**
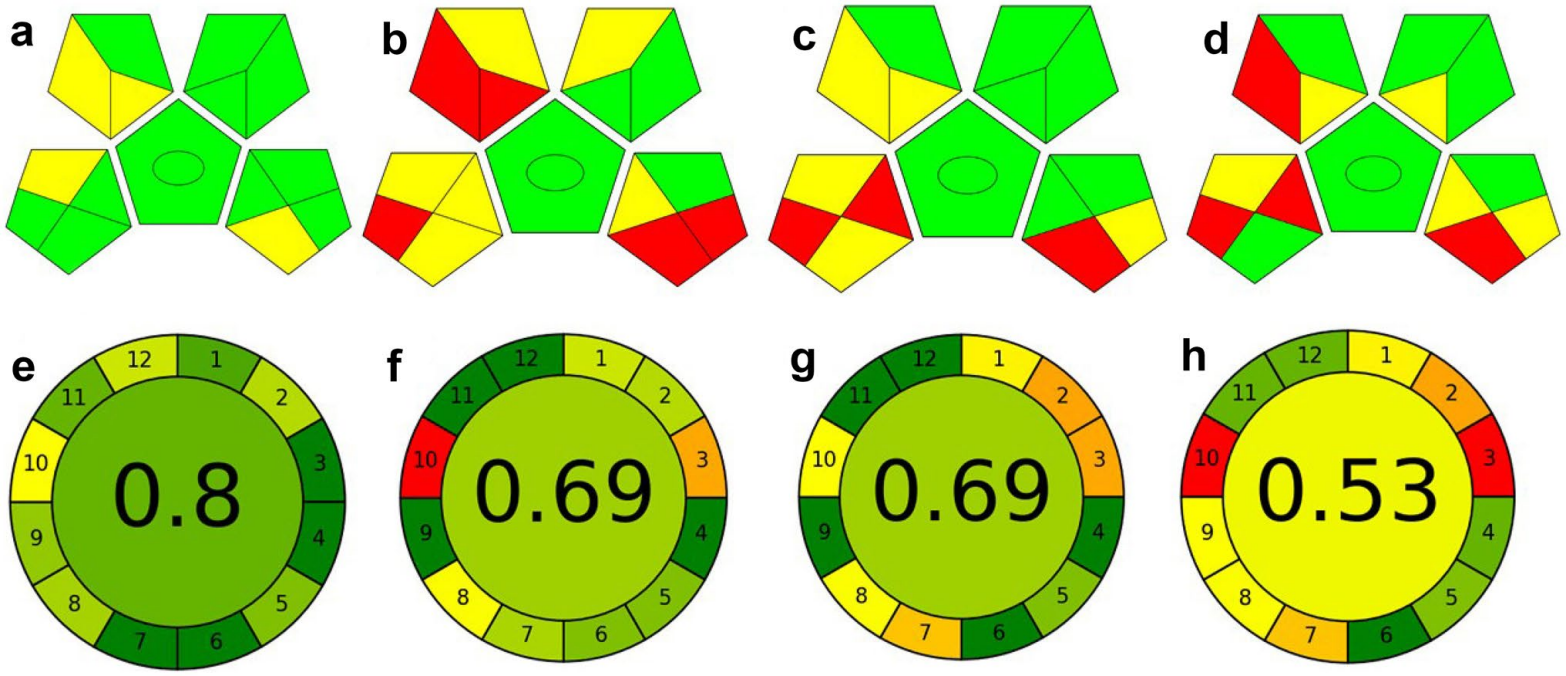
Pictograms used for the greenness assessment using GAPI and AGREE tools for **a**, **e** the proposed method, and previously reported methods **b**, **f** for Ref. 3, **c**, **g** for Ref. 8, and **d**, **h** for Ref. 17, respectively. In GAP index, the red shades represent high ecological impact, yellow shades represent lower impact, and the green ones represent the safe mode

GAPI is a semi-quantitative tool based on evaluating all aspects of analytical methods, such as sample collection, preservation, transport, storage, sample preparation and final analysis. It measures the greenness of the method semi-quantitatively through formation of colored-shaded pentagrams subdivided into fifteen smaller sections. The colors give an indication of the degree of each aspect from low-medium to high impact (green-yellow to red) [51]. Upon comparing the proposed method with three previously reported procedures for determination of Ara-C, their GAPI pictograms are shown in Fig. 7. It can be seen that the proposed procedure (Fig. 7a) contains eleven green-shaded zones (11) and four yellow pentagrams (4), with the absence of red zones. On the other hand, the previously reported methods (b, c, d) contain 5, 3, and 4 red zones, indicating high ecological impact.

AGREE is another semi-quantitative evaluation tool based on the twelve green analytical chemistry (GAC) principles. It is represented as a clock-shaped pictogram divided into 12 sections; each section inferred to one of the GAC principles. The analytical greenness score is displayed in the middle, ranging from 0.0 (the lowest score) to 1.0 (perfect score).

Each aspect in AGREE tool is evaluated at three levels and provides more information about the energy consumption, and the environmental hazards [52]. As shown in Fig. 7, AGREE of the presented method shows low ecological impact as it expressed by 0.8 value (Fig. 7e). It has better profile regarding the waste amount, except for the source of the reagent. The use of low energy fluorimetric method and the simplicity in the preparation of sample without the need of any derivatizing agent make the proposed method of green environmental behavior.

## Conclusion

In this study, modified graphene quantum dots with ZnO has been successfully developed and utilized as fluorimetric platform for highly sensitive and selective determination of Ara-C. The determination protocol is based on “Turn Off/On” strategy of the strong native fluorescence of the prepared ZnO-QDs which is turned Off by Ce^4+^ ions as a quencher with a wide range of 2.5 to 30 μmol L^−^^1^. After the addition of ethylenediamine as a linker into Ce@ZnO-QDs system, the obtained ED/Ce@ZnO-QDs exhibit high complexation power towards Ara-C. Upon the addition of Ara-C to the quenched ED/Ce@ZnO-QDs, the FL intensity was clearly restored due to the formation of Ara-C/ED/Ce compound and the release of the fluorimetric probe ZnO-QDs (Turn On). The developed nanoprobe was successfully detected Ara-C in the concentration range of 10 to 1000 ng mL^−1^ (LOD = 2.87 ng mL^−1^). The developed sensing probe provided excellent selectivity towards different metals and some interfering substances. Additionally, the proposed sensing nanoprobe was successfully applied for analysis of Ara-C in pure and pharmaceutical dosage forms and in spiked human plasma, and the recoveries were in the range of (99.02 ± 0.479%) to (101.71 ± 2.02%). The developed nanoprobe was utilized to study the pharmacokinetic behavior of Ara-C after I.V. injection in rabbits. In order to increase the sensitivity of the proposed method and to eliminate the interference in the complicated biological samples, dispersive micro-extraction solid phase technique was applied using thiol-modified magnetic nanoparticles. Additionally, the adsorption behavior of Ara-C on the developed nanoprobe was studied. The adsorption experiments showed the adsorption capacity increased with increasing contact time, and the adsorption mechanism followed pseudo-second adsorption and Langmuir adsorption models as adsorption kinetics and isotherm models, respectively. Additionally, the greenness evaluation of the proposed method was achieved using two scores; GAPI and AGREE tools. The results concluded that the proposed method is green, environmentally safe and with no potential hazards compared with some previously reported methods.

## Supplementary Information

Below is the link to the electronic supplementary material.

Supplementary file1 (DOCX 451 KB)

## Data Availability

Not applicable.
