# Correction: “Off/On” Fluorescent Probe based on Aggregation-Induced Quenching of ZnO-Quantum dots for Determination of Ara-C: Pharmacokinetic Applications, Adsorption Kinetics & Green Profile Assessment

**DOI:** 10.1007/s10895-025-04351-6

**Published:** 2025-05-02

**Authors:** Marwa R. El-Zahry, Rania S. Ibrahim, Hanaa M. Abd El-Wadood, Horria A. Mohamed

**Affiliations:** 1 Pharmaceutical Analytical Chemistry Department, Faculty of Pharmacy, Assiut University, Assiut, 71526 Egypt; 2 Pharmaceutical Chemistry Department, Faculty of Pharmacy, Badr University in Assiut, Assiut, 2014101 Egypt

**Correction: Journal of Fluorescence (2023) 34:1617–1630**

10.1007/s10895-023-03359-0

In this article, the ‘Ethical Approval’ statement was incorrectly given as ‘The research protocol of animal studies was reviewed and approved by the Institutional Animal Ethical Committee of the Faculty of Medicine (No. AEC04363229), Assiut university, Assiut, Egypt’ but should have been ‘The research protocol of animal studies was approved by the Ethical Committee of the Faculty of Veterinary Medicine, Assiut University, Assiut, Egypt’ according to The OIE standards for use of animals in research, under the No. 06/2023/0050'.

The original article has been corrected.

