# Supplementary material for: "Off/On” Fluorescent Probe based on Aggregation-Induced Quenching of ZnO-Quantum dots for Determination of Ara-C: Pharmacokinetic Applications, Adsorption Kinetics & Green Profile Assessment
Source: J Fluoresc. 2023 Aug 11;34(4):1617–30. doi: 10.1007/s10895-023-03359-0 (PMC11249414; doi:10.1007/s10895-023-03359-0)
Supplement: Supplementary file 1 — Supplementary file1 (DOCX 451 KB) [file 10895_2023_3359_MOESM1_ESM.docx]

**Electronic Supplementary Materials (ESM)**

**“Off/On” Fluorescent Probe based on Aggregation-Induced Quenching of ZnO-Quantum dots for Determination of Ara-C: Pharmacokinetic Applications, Adsorption Kinetics & Green Profile Assessment**

**Marwa R. El-Zahry^1,2^*, Rania S. Ibrahim^1^, Hanaa M. Abd El-Wadood^1^, Horria A. Mohamed^1^**

^1^ Pharmaceutical Analytical Chemistry Department, Faculty of Pharmacy, Assiut University, Assiut 71526, Egypt

^2^ Pharmaceutical Chemistry Department, Faculty of Pharmacy, Badr University in Assiut, Assiut 2014101, Egypt

***Corresponding author:**

Address: Department of Pharmaceutical Analytical Chemistry, Faculty of Pharmacy, Assiut University, 71526 Assiut, Egypt

Email: marwazahry@aun.edu.eg

Fax number: 0020-88-2080774

**Application of the proposed method**

### **Pharmaceutical dosage form**

One milliliter of Tabine ampoule (100 mg 5mL^-1^) was transferred into a 100 mL volumetric flask and completed to the mark with distilled water. Further dilutions were made to obtain a working solution of 2 µg mL^-1^ and the analysis procedure was conducted as described under fluorescence sensing procedure.

### **Spiked human plasma**

Into a 10 mL-centrifuge tube, 1 mL of human plasma free from drug was added to 1 mL of 100 ng mL^-1^ Ara-C solution. Three milliliters of acetonitrile were added and centrifuged at 10000 rpm for 30 min. The clear supernatant was transferred into a 10 mL volumetric flask and the analysis procedure was processed as described under magnetic solid-phase extraction procedure and fluorescence sensing procedure.

## **Pharmacokinetic study**

The proposed probe was utilized for the pharmacokinetic study of Ara-C in rabbits plasma samples. Three male rabbits (Bauscat rabbits) were judged healthy based on physical examination and weighing (2.0 ± 0.3 kg).

Rabbits were housed under 12 hr light/dark cycle in a temperature- and humidity- controlled room (15-25^°^C, 55-65% relative humidity) and allowed water and standard laboratory chow *ad libitum*. The room was spot cleaned daily and deep cleaned twice weekly. The animal dose was extrapolated from human one using a previously reported equation [1], which considers the differences in body surface area and pharmacokinetics among different species. Each rabbit was injected with single intravenous dose that is equivalent to 17 mg kg^-1^ from its pharmaceutical dosage form.

The blood samples were collected at pre-determined intervals from retro-orbital venous plexus using heparinized capillary tubes, followed by centrifugation for 10 min. at 4000 rpm. The separated plasma samples were stored at -20^°^C and analyzed using the developed fluorimetric sensing probe. Different pharmacokinetic parameters including elimination half-life (t_0.5_), clearance (CL), volume of distribution (V_d_), area under the plasma concentration-time curve (AUC_0-t_) were estimated using Kinetica 5.0 software.

**Optimization of parameters affecting the developed system on human plasma**

Different variables affecting the application of the proposed method in human plasma were investigated and described in Fig. S5.

#### **Type and volume of protein precipitating agent**

Various types and volumes of protein precipitating agents were investigated. Acetonitrile with a volume of 3 mL was selected for complete precipitation of plasma proteins.

**Speed of centrifugation**

Different speeds of centrifugation over the range 4,000 to 14,000 rpm have been studied. It was found that recovery percentage increases from 4,000 rpm to 10,000 rpm then begins to remain constant until 14,000 rpm. Hence, the optimum speed of centrifugation was selected to be 10,000 rpm.

#### **Effect of time of centrifugation**

The effect of different times of centrifugation from 10 min. up to 60 min. was investigated on the precipitation of spiked plasma samples with Ara-C using acetonitrile as a precipitating agent at 10,000 rpm. It was found that by increasing time of centrifugation there is an increase in % recovery of Ara-C up to 30 min. then it remains constant up to 60 min. So, the optimum time of centrifugation was 30 min.

#### **Validation of spiked human plasma**

#### **Linearity, detection and quantitation Limits**

A linear relationship between FL intensity and Ara-C concentration was obtained over the range 10 to 1000 ng mL^-1^ with LOD and LOQ of 3.19 and 9.61 ng ml^-1^, respectively. Different statistical parameters were calculated and listed in Table S1.

**
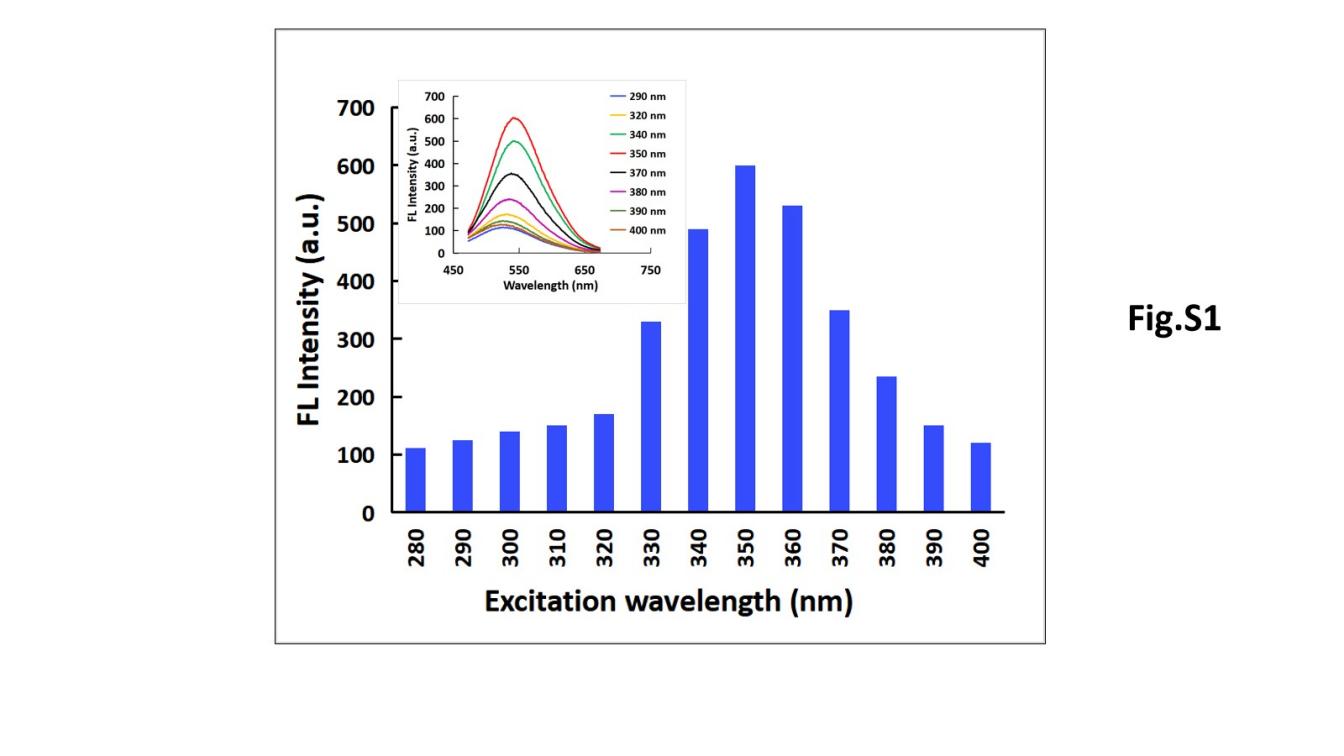
**

**Fig. S1** Effect of excitation wavelength on the FL intensity of 500 ng mL^-1^ Ara-C emitted at 532 nm. Inset: Emission spectra of 500 ng mL^-1^ Ara-C excited at different excitation wavelengths

**
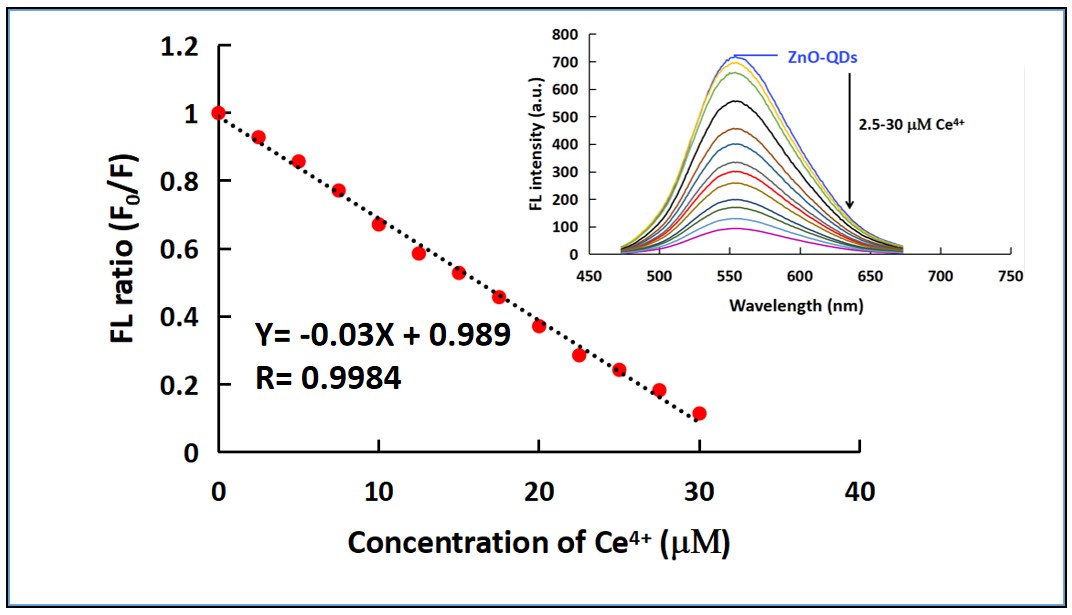
**

**Fig. S2** Calibration of Ce^4+^ (2.5- 30 μM) by quenching of ZnO-QDs at ʎ_exc._ =350 nm and ʎ_em_ =532 nm

**
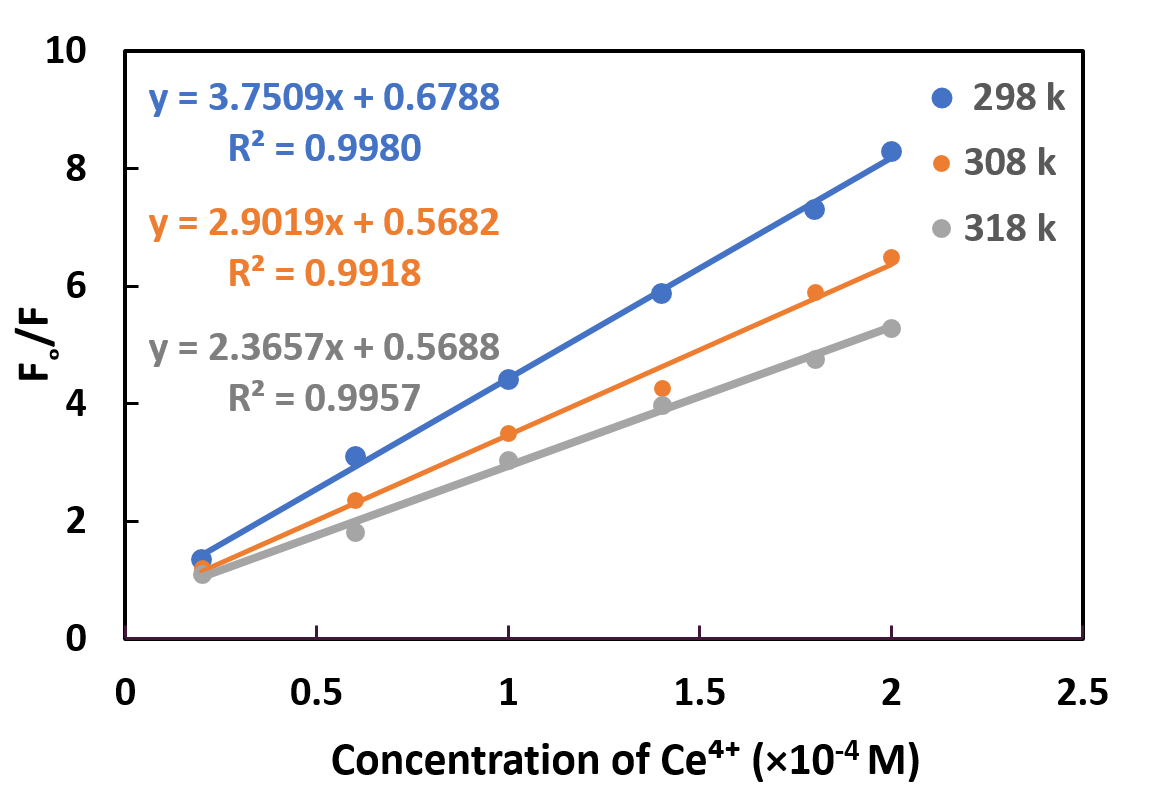
**

**Fig. S3** Stern Volmer curves of Ce^+4^ quenched ZnO-QDs at different temperatures (298, 308 and 318 K)

**
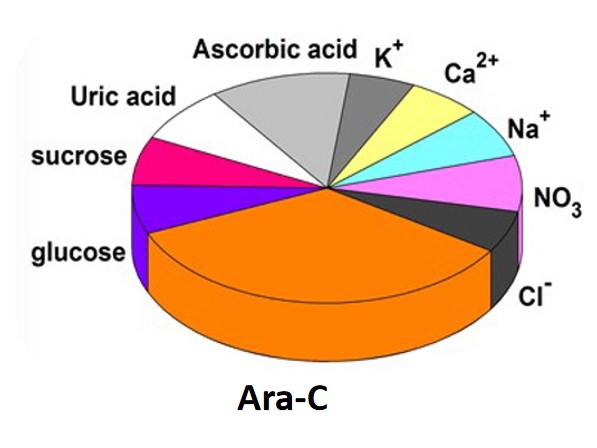
**

**Fig. S4** Pie chart representing the selectivity of Ara-C on the proposed platform using ED/Ce@ZnO-QDs and different concentrations of biologically interfering compounds

**
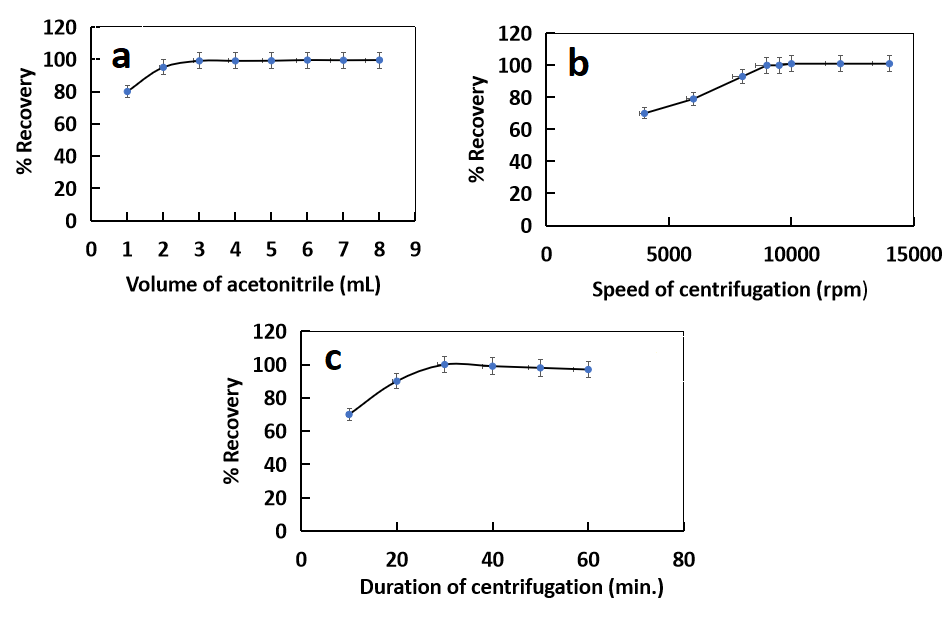
**

**Fig. S5** The effect of (a) volume of acetonitrile, (b) speed of centrifugation, (c) duration of centrifugation on % recovery of 100 ng mL^-1^ Ara-C spiked in human plasma sample using the proposed probe and 2 mL of 10^-3^ mol L^-1^ Ce^4+^ at ʎ_exc._ = 350 nm and ʎ_em_ =532 nm

**
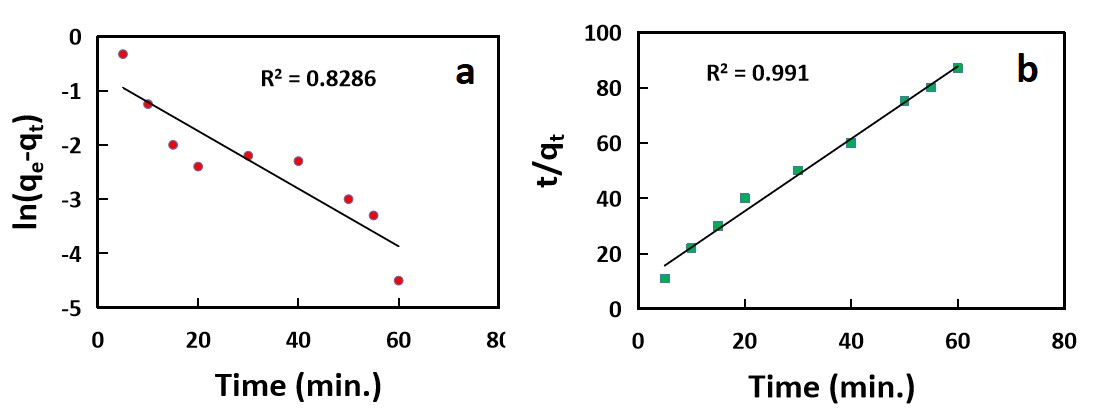
**

**Fig. S6** Linearized pseudo first-order (a) and pseudo second order (b) equation plot for adsorption of Ara-C onto S-MNPs

**
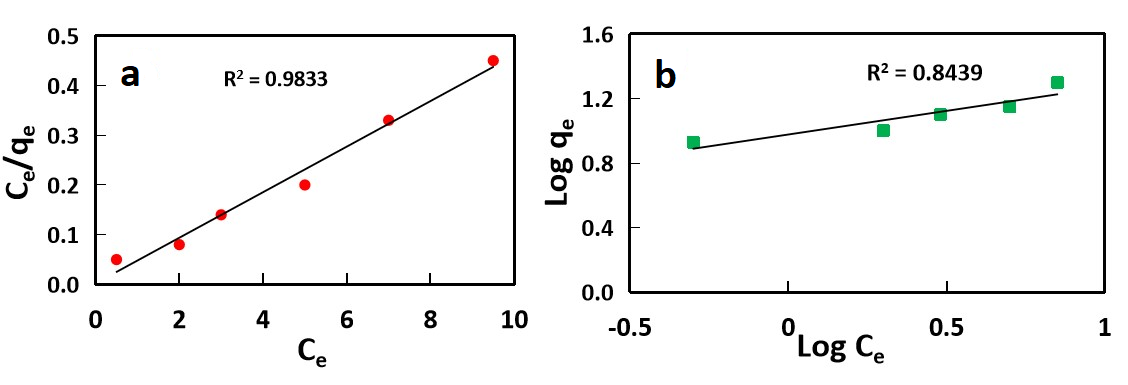
**

**Fig. S7** Linear Langmuir (a) and Freundlich (b) adsorption isotherms for adsorption of Ara-C on S-MNPs

**Table S1** Robustness of the developed method

| **Parameter** | **% Recovery* ± SD** | **RSD %** |
| --- | --- | --- |
| No variation ^**^ | 100.33 ± 0.558 | 0.552 |
| Concentration of ZnO-QDs  (Optimum value = 2.5 ×10^-3^ mg mL^-1^)  2.0 ×10^-3^ mg mL^-1^  3.0 ×10^-3^ mg mL^-1^ | 99.26 ± 0.53  102.13 ± 0.70 | 0.547  0.703 |
| Concentration of Ce ^4+^  (Optimum value = 1.5 μmol L^-1^)  1.0 μmol L^-1^  2.0 μmol L^-1^ | 98.66 ± 0.61  99.91 ± 1.31 | 0.621  1.314 |
| Excitation wavelength  (Optimum value = 350 nm)  348 nm  352 nm | 100.71 ± 0.54  101.88 ± 0.77 | 0.536  0.755 |

*Average of six replicates.

**No variation in the experimental conditions of the proposed method.

**Table S2** Kinetic parameters for the pseudo-first-order and pseudo-second-order models

| **Model order of kinetic** | **Parameters** | | |
| --- | --- | --- | --- |
|  | Rate constant | q_e_ (mg g^-1^) | R^2^ |
| **Pseudo first order** | 0.0560 | 0.2352 | 0.8286 |
| **Pseudo second order** | 0.8416 | 1.012 | 0.9910 |

**Table S3** Langmuir and Freundlich isotherm model parameters for the adsorption of Ara-C on S-MNPs

| **Isotherm model** | **Parameters** | | |
| --- | --- | --- | --- |
| **Langmuir model** | q_max_ (mg g^-1^) | k_L_ (L mg^-1^) | R^2^ |
|  | 40.1 | 0.99 | 0.9833 |
| **Freundlich model** | 1/n | k_F_ (L mg^-1^) | R^2^ |
|  | 0.34 | 5.6 | 0.8439 |

**References**

[1] Nair A, Jacob S (2016) A simple practice guide for dose conversion between animals and human, J Basic Clin Pharm. 7:27. https://doi.org/10.4103/0976-0105.177703.
